# Walking Outcome After Traumatic Paraplegic Spinal Cord Injury: The Function of Which Myotomes Makes a Difference?

**DOI:** 10.1177/15459683231166937

**Published:** 2023-04-11

**Authors:** Adrian Cathomen, Doris Maier, Jiri Kriz, Rainer Abel, Frank Röhrich, Michael Baumberger, Giorgio Scivoletto, Norbert Weidner, Rüdiger Rupp, Catherine R. Jutzeler, John D. Steeves, EMSCI study group, Armin Curt, Marc Bolliger

**Affiliations:** 1 Spinal Cord Injury Center, Balgrist University Hospital, Zurich, Switzerland; 2 ETH Zurich, Zurich, Switzerland; 3 Neuroscience Center Zurich, University of Zurich, Zurich, Switzerland; 4 Trauma Center Murnau, Murnau, Germany; 5 Spinal Cord Unit, University Hospital Motol, Prague, Czech Republic; 6 Berufsgenossenschaftliche Kliniken Bergmannstrost, Zentrum für Rückenmarkverletzte und Klinik für Orthopädie, Halle, Germany; 7 Swiss Paraplegic Center, Nottwil, Switzerland; 8 Spinal Unit and Spinal Rehabilitation (SpiRe) Lab, IRCCS Fondazione S. Lucia, Rome, Italy; 9 Spinal Cord Injury Center, Heidelberg University Hospital, Heidelberg, Germany; 10 Biomedical Data Science Lab, Department of Health Sciences and Technology, ETH Zurich, Zurich, Switzerland; 11 Schulthess Clinic, Zurich, Switzerland; 12 ICORD, University of British Columbia and Vancouver Coastal Health, Blusson Spinal Cord Centre, Vancouver, BC, Canada

**Keywords:** spinal cord injury, walking function, unbiased recursive partitioning, stratification, muscle function, myotomes, rehabilitation

## Abstract

**Background:**

Accurate prediction of walking function after a traumatic spinal cord injury (SCI) is crucial for an appropriate tailoring and application of therapeutical interventions. Long-term outcome of ambulation is strongly related to residual muscle function acutely after injury and its recovery potential. The identification of the underlying determinants of ambulation, however, remains a challenging task in SCI, a neurological disorder presented with heterogeneous clinical manifestations and recovery trajectories.

**Objectives:**

Stratification of walking function and determination of its most relevant underlying muscle functions based on stratified homogeneous patient subgroups.

**Methods:**

Data from individuals with paraplegic SCI were used to develop a prediction-based stratification model, applying unbiased recursive partitioning conditional inference tree (URP–CTREE). The primary outcome was the 6-minute walk test at 6 months after injury. Standardized neurological assessments ≤15 days after injury were chosen as predictors. Resulting subgroups were incorporated into a subsequent node-specific analysis to attribute the role of individual lower extremity myotomes for the prognosis of walking function.

**Results:**

Using URP–CTREE, the study group of 361 SCI patients was divided into 8 homogeneous subgroups. The node specific analysis uncovered that proximal myotomes L2 and L3 were driving factors for the differentiation between walkers and non-walkers. Distal myotomes L4–S1 were revealed to be responsible for the prognostic distinction of indoor and outdoor walkers (with and without aids).

**Conclusion:**

Stratification of a heterogeneous population with paraplegic SCI into more homogeneous subgroups, combined with the identification of underlying muscle functions prospectively determining the walking outcome, enable potential benefit for application in clinical trials and practice.

## Introduction

Recovery of walking function is among the outcomes with the highest priority for individuals with a paraplegic spinal cord injury (SCI).^1^ Accordingly, locomotor training claims a significant share during rehabilitation in clinical practice and walking function is targeted by various therapeutical interventions in clinical trials.^2-5^ To determine whether a patient has the potential to regain ambulation throughout rehabilitation and to further provide an accurate and reliable prognosis on the walking outcome is of crucial importance for an appropriate tailoring of rehabilitation interventions. In addition, accurate outcome prediction serves as valuable information for health professionals and patients to manage expectations.

However, providing an evidence-based rationale for the prognosis of walking function represents a complex task in SCI, a neurological disorder presented with heterogeneous clinical manifestations and recovery trajectories.^6^ Over the recent years, many prediction models targeting walking function have been developed.^7-11^ However, they use dichotomous outcomes, potentially missing valuable information granted when considering a continuous endpoint. A continuous endpoint, on the other hand, allows for the identification of additional and more homogeneous subgroups.^12^ To address this issue, we applied a novel method of stratification for a continuous outcome in individuals with cervical SCI.^12^

With regard to the prognosis of ambulation, literature attributes significant importance to the muscle function in determining the outcome in paraplegia.^13,14^ Further analysis of lower extremity myotomes and their related key muscles revealed either L2 (hip flexion), L3 (knee extension), or a combination thereof to be the driving factor(s) for walking function.^15-18^ Others suggest slightly modified combinations (L3 and L5 or S1).^8,19^ These studies provide a valuable basis to build on, however, they are often limited regarding either the sample size, the availability of longitudinal data, or low sensitivity owing to a dichotomous outcome variable.

To determine the factors that predict walking function after traumatic paraplegic SCI and assign the role of the individual lower extremity myotomes in this context, we introduce an approach relying on a prediction-based stratification model. Predictor data ≤15 days after injury were assessed to stratify walking function at 6 months after injury, using the continuous outcome of the 6-minute walk test (6MWT),^20^ with the aim of identifying more homogeneous subgroups. For this purpose, the technique of unbiased recursive partitioning conditional inference trees (URP–CTREE) was applied.^21^ To verify the influence of the individual lower extremity myotomes on the outcome of ambulation, we made use of the subgroups stratified by the statistical model and investigated the muscle function in myotomes L2–S1 at ≤15 days and 6 months after injury within each subgroup.

## Methods

### Data Source

The analysis relies on data extracted from the European Multicenter Study about Spinal Cord Injury (EMSCI; ClinicalTrials.gov Identifier: NCT01571531). EMSCI spans a network of SCI centres, which prospectively collect data from individuals after acute SCI over the first year of injury. Applying a standardized assessment protocol, the patients’ neurological and functional condition is collected ≤15 days and 1, 3, 6, and 12 months after injury. The EMSCI network has acquired ISO–9001:2015 certification, and professionals are trained to properly conduct all required assessments to guarantee high quality levels of the recorded data.^22^

Data of adults (≥18 and ≤70 years) with acute traumatic paraplegic (neurological level of injury from T2 to S1) SCI, enrolled in EMSCI between August 2001 and July 2019, were extracted from the database. The study has approval of the local ethics committees of participating centres and was completed according to the Declaration of Helsinki.

### Predictors and Outcomes

For the stratification model, we considered neurological and demographic predictors (assessed ≤15 days after injury) and functional outcomes (assessed 6 months after injury), based on previous findings^23^ and according to clinical research interests in rehabilitation of ambulation. The following assessments of the International Standards for Neurological Classification of Spinal Cord Injury^24^ (ISNCSCI) were included as predictors: the single NLI, total lower extremity motor score (LEMS), and total sensory scores (light touch [LT] and pin prick [PP] sensation). Additionally, age at injury was included as a covariate.

As primary outcome for this study, we selected the 6MWT at 6 months after injury to assess walking function using an established outcome measure featuring a continuous scale. Additionally, the following outcome measures at 6 months after injury were included for analysis: 10-m walk test (10 mWT),^25^ Spinal Cord Independence Measure III (SCIM III) mobility items 12 to 14 (item 12, mobility indoors; item 13, mobility for moderate distances [10-100 m]; and item 14, mobility outdoors [≥100 m]; hereafter referred to as SCIM_12-14_),^26^ Walking Index for Spinal Cord Injury (WISCI II),^27^ and LEMS.

The subgroups identified with the stratification model were categorized in terms of walking function based on their outcome and applicability in daily living to obtain distinct and meaningful functional levels of ambulation. We classified patients according to their walking speed of 10 mWT (adapted from van Hedel et al^28^) at 6 months after injury: *non*-*walkers* (10 mWT = 0 m/s), *indoor walkers* (0 < 10 mWT ≤ 0.54 m/s; able to walk indoors, but wheelchair-dependent outdoors), *outdoor walkers* (10 mWT > 0.54 m/s; able to walk indoors and outdoors). The outdoor walker group was further divided based on the SCIM III_14_ at 6 months after injury: *outdoor walkers with aids* (SCIM III_14_ < 8; dependent on walking aids to walk outdoors) and o*utdoor walkers without aids* (independent of walking aids to walk outdoors) to provide a more refined categorization in the group with the least walking impairment. Hence, 4 categories of walking function were specified.

### Patient Population

For the analysis, a total of 879 patients from 29 SCI centres of the EMSCI network were extracted from the database and divided into 2 groups: (i) a *study group* and (ii) a *reference group* (eFigure 1).

The study group (N = 361) served as basis for the development of the stratification model and subsequent analyses. Only subjects with a complete documentation of predictors ≤15 days and outcomes at 6 months after injury were included.

The reference group (N = 518) was used to verify the representativeness of the study group within the EMSCI database at the acute stage of injury. Required for inclusion were complete predictor data ≤15 days after injury, without the availability of all outcomes at 6 months after injury.

Data was checked for sanity prior to analysis, explained in detail in the supplemental material. The occurrence of missing data is known to vary with severity of injury in SCI and other pathologies.^29,30^ Injury severity is classified using the American Spinal Injury Association Impairment Scale (AIS) grades A to E (eTable 1).^24^ Hence, we compared the study and reference groups for each AIS grade (ie, A-D) for LEMS, LT, and PP ≤15 days after injury.

### Stratification Model

Based on the study group we developed a stratification model, applying URP–CTREE (a tree-based regression analysis), aiming to split a heterogeneous group into several more homogeneous subgroups. In this heterogeneous group, URP–CTREE searches the predictor with the strongest statistically significant association with the selected outcome, enabling a splitting of the group into 2 maximally distinct subgroups (with respect to the outcome). The model thereby adjusts for multiple comparisons and deals with collinearity. This procedure is repeated until no further statistically significant association between any predictor and outcome can be found. The result is a set of *nodes* (subgroups of the initial group) and a simple tree-based stratification rule to allocate prospective patients to the corresponding nodes. More information on the basic theoretical framework is provided in Hothorn et al.^21^ Reliability and robustness of URP–CTREE in stratification and prediction of outcomes in SCI has recently been demonstrated.^31^

### Lower Extremity Myotomes

The LEMS is a composite score comprising five muscles (per leg) representing five consecutive myotomes. A myotome is defined as a group of muscles innervated from the same nerve root. According to ISNCSCI, the myotomes of the lower extremity key muscle functions are innervated from the spinal levels L2 to S1. Motor scores are assessed for each key muscle function of a myotome (graded from 0 to 5) and added up to the LEMS (eTable 2).^24^ To determine the neurological status and recovery of motor function, we divided the LEMS in its individual myotomes and analyzed the data for the more (MI) and less impaired (LI) leg at the timepoints ≤15 days and 6 months after injury for each node of the URP–CTREE. Most injuries among patients with residual muscle function in the lower extremities are not side-symmetrical, implying differences in condition and recovery between the 2 legs. MI and LI leg were defined according to (in order of precedence): total LEMS (at 6 months and ≤15 days after injury) and motor scores of individual lower extremity myotomes (at 6 months and ≤15 days after injury) at levels L2–S1 of the corresponding side. The leg with the higher score was defined as the LI leg and the leg with the lower score was defined as the MI leg, respectively. If none of the criteria allowed for a definition of MI and LI leg, the right side was defined as MI and the left side as LI, respectively. Statistical analysis focusing on the myotomes L2–S1 was performed between the 4 specified categories of walking function, as they represent distinct and meaningful functional levels of ambulation.^28^

### Statistics

The tree-based regression analysis (URP–CTREE) was performed in the computing environment R (version 4.0.4, Windows),^32^ using the statistical package *party* (version 1.3-10).^33^ Further statistical analyses were conducted in R and SPSS (V27, IBM Corp., USA).

### Data and Code Availability

The data used for this study, including anonymized individual participant data and a data dictionary defining each field or variable within the dataset, can be made available on reasonable request to the corresponding author (MB). Written proposals will be evaluated by the authors, who will render a decision regarding suitability and appropriateness of the use of data. Approval of all authors and the EMSCI consortium will be required, and a data sharing agreement must be signed before any data are shared. The code to run the analysis can be found on our github repository (https://github.com/adriancathomen/URP-CTREE-analysis-paraplegic-patients.git). The study protocol and a detailed description of the project is accessible on the official website of the EMSCI network (https://www.emsci.org).

## Results

### Study Population

The baseline characteristics of study and reference groups are reported in Table 1. Patients were mostly of male sex (∼80%), around 40 years of age at injury, and predominantly with an injury on a thoracic level (81% in the study group and 74% in the reference group). The proportion of patients graded as AIS A was greater in the study group, whereas the reference group contained more AIS C and D patients. Accordingly, we observed slightly higher scores for LEMS, LT, and PP in the reference group. However, comparing the 2 groups stratified by AIS grades revealed similar LEMS, LT, and PP scores across all AIS grades (eFigure 2).

**Table 1.** Group Characteristics.

	Study group N = 361	Reference group N = 518
Sex		
Male	287 (79.5%)	418 (80.7%)
Female	74 (20.5%)	100 (19.3%)
Age (years)		
Mean ± SD	40.4 ± 14.4	38.7 ± 13.7
Neurological level of injury		
T2-12	291 (80.6%)	382 (73.8%)
L1-5	70 (19.4%)	134 (25.9%)
S1	0 (0.0%)	2 (0.4%)
AIS grades		
A	223 (61.8%)	277 (53.5 %)
B	48 (13.3%)	68 (13.1%)
C	40 (11.1%)	77 (14.9%)
D	50 (13.9%)	96 (18.5%)
Lower extremity motor score		
Median (IQR)	0 (0-13)	1 (0-22)
Light touch		
Median (IQR)	75 (56-94)	80 (62-99)
Pin prick		
Median (IQR)	73 (54-90)	78 (59-96)

Abbreviations: AIS, American Spinal Injury Association impairment scale; IQR, interquartile range; LEMS, lower extremity motor score; LT, light touch; N, number of patients; NLI, neurological level of injury; PP, Pin prick; SD, standard deviation.

Characteristics of study and reference groups. Baseline predictor data (age, NLI, LEMS LT, and PP) and AIS are recorded ≤15 days after injury. Further comparison of lower extremity motor score, light touch, and pin prick between groups, divided into AIS grades, is provided in eFigure 1.

### Stratification Model

**Figure 1.**
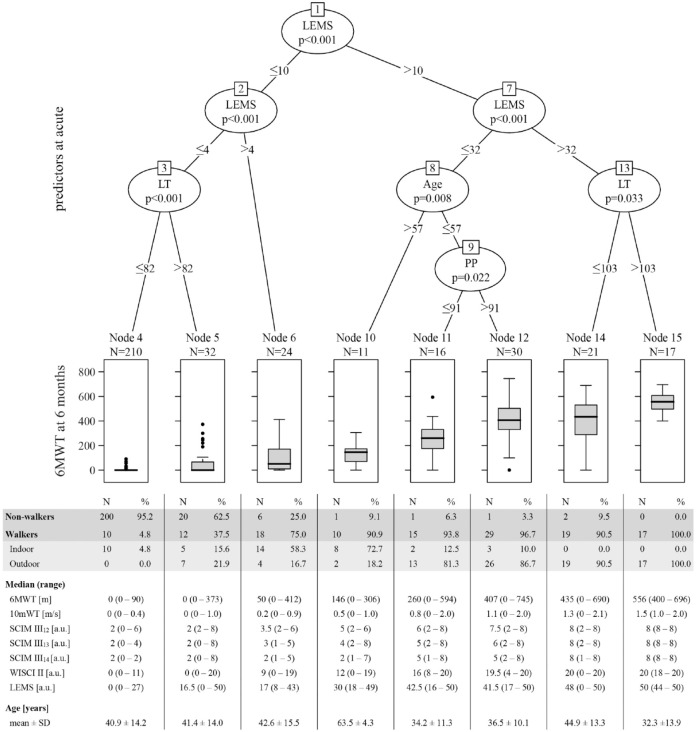
Stratification model for the 6MWT for individuals (study group; N = 361) with a thoracolumbar SCI. URP–CTREE for 6MWT at 6 months after injury (displayed in boxplots with median and Tukey whiskers), stratified by the predefined predictors (age, lower extremity motor score [LEMS], light touch [LT], neurological level of injury [NLI], and pin prick [PP]) characterizing the neurological and functional status of patients ≤15 days (acute) after injury. Distribution of non-walkers and walkers (indoor and outdoor) within the nodes of the 6MWT URP–CTREE is presented. Median and range of 6MWT, 10 mWT, SCIM III_12-14_, WISCI II, and LEMS outcomes at 6 months after injury, and age are reported for each node of the URP–CTREE. Distribution of NLI and AIS in each node of the URP–CTREE is provided in eTable 3. Significance level at *P* < .05. Abbreviations: 6MWT, 6-minute walk test; 10 mWT, 10-m walk test; a.u., arbitrary unit; SCI, spinal cord injury; SCIM III_12-14_, spinal cord independence measure III item 12 (mobility indoors), item 13 (mobility for moderate distances [10-100 m]), and item 14 (mobility outdoors [≥100 m]); URP–CTREE, unbiased recursive partitioning conditional inference tree; WISCI II, walking index for spinal cord injury.

With the application of URP–CTREE and the use of predictor data ≤15 days after injury, we developed a stratification model for the 6MWT at 6 months after injury (Figure 1). The study group was divided, with regard to the 6MWT, into eight terminal nodes (# 4, 5, 6, 10, 11, 12, 14, and 15), according to the splits at the inner nodes (# 1, 2, 3, 7, 8, 9, and 13). The stratification model selected LEMS, LT, age, and PP as the strongest outcome–associated predictors on the respective level of the URP–CTREE. Distribution of the 6MWT outcome at 6 months after injury in the resulting nodes was characterized by a gradient from no to very good walking performance. Nodes 4 and 5 (non-walkers) showed no to almost no ambulation capacity, whereas nodes 6 and 10 (indoor walkers) revealed poor walking performances, and nodes 11 to 15 (outdoor walkers) were reported with moderate to very good outcomes in the 6MWT.

Preserved lower extremity muscle function ≤15 days after injury (LEMS >4) appears to separate, on group level, walkers from non-walkers at 6 months after injury. Generally, higher scores in LEMS in combination with higher scores in LT and PP at baseline, describing lower injury severity, together with young age, favour better outcomes in walking function.

Figure 1 further provides information on the proportion of walkers and non-walkers in each node, as well as additional outcome measures related to walking function to better characterize the individual nodes (with regard to speed [10 mWT], ability to walk different distances in daily life [SCIM III_12-14_] and the corresponding use of walking aids and orthoses [WISCI II], and muscle function [LEMS]).

NLI and AIS grades of patients within each node of the URP–CTREE are reported in eTable 3. For both measures, we did not find clear distribution patterns among nodes, but rather unspecific occurrences, except for nodes 4 and 15.

### Lower Extremity Myotomes

**Figure 2.**
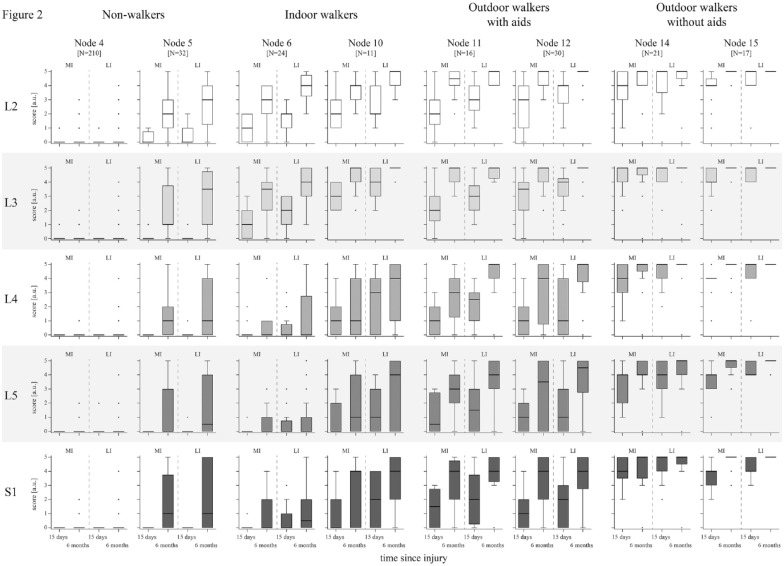
Neurological status and recovery within the nodes of the 6MWT URP–CTREE. LEMS is divided in its component parts (5 myotomes on the spinal cord levels L2–S1). Key muscle function according to the myotomes is displayed for every node and divided into MI and LI leg at the timepoints ≤15 days and 6 months after injury. Data are shown as boxplots (whiskers: Tukey) for the categories of non-walkers (nodes 4 and 5), indoor walkers (nodes 6 and 10), outdoor walkers with aids (nodes 11 and 12), and outdoor walkers without aids (nodes 14 and 15). Statistical analysis between the 4 walking categories is provided in Table 2. A congruent figure, extended by dotted lines connecting the individual patient values between the 2 timepoints, can be found in eFigure 3. Abbreviations: 6MWT, 6-minute walk test; a.u., arbitrary unit; L2, myotome of hip flexors; L3, myotome of knee extensors; L4, myotome of ankle dorsiflexors; L5, myotome of long toe extensors; LEMS, lower extremity motor score; LI, less impaired; MI, more impaired; N, number of patients; S1, myotome of ankle plantar flexors; URP–CTREE, unbiased recursive partitioning conditional inference tree.

Based on the nodes of the URP–CTREE, we analyzed the individual muscle groups of the more and less impaired side (myotomes L2–S1) to display the neurological status and recovery of motor function (Figure 2). Overall, muscle function in proximal myotomes L2 and L3 was characterized in a gradually increasing manner, according to the nodes’ walking performance, from no (node 4) to excellent function (node 15). Likewise, the pattern of residual and recovered muscle function in the distal myotomes L4–S1 was displayed.

Statistical analysis of myotomes of the MI leg ≤15 days after injury between the 4 specified categories of walking function at 6 months after injury is presented in Table 2. This analysis revealed highly significant differences in proximal myotomes L2 (hip flexion) and L3 (knee extension) between non-walkers and indoor walkers. The group of non-walkers showed no muscle function early after injury in any myotome (motor score median [range] = 0 [0-1]), compared to indoor walkers with residual muscle function in myotomes L2 (motor score median [range] = 1 [0-5]) and L3 (motor score median [range] = 2 [0-4]). However, proximal myotomes were not able to distinguish between the 3 categories of walkers (indoor vs outdoor with aids, and outdoor with aids vs outdoor without aids). The motor functions in distal myotomes L4 (ankle dorsiflexion), L5 (long toe extension), and S1 (ankle plantarflexion) were significantly different between these walking categories. Indoor walkers were thereby characterized by a lack of muscle function of the MI leg in distal myotomes L4, L5, and S1 (motor score median [range] = 0 [0-4]) ≤15 days after injury. Significantly more motor function was preserved in outdoor walkers with aids in distal myotomes L4 (motor score median [range] = 1 [0-4]), L5 (motor score median [range] = 1 [0-3]), and S1 (motor score median [range] = 1 [0-4]). The same pattern was observed when comparing the 2 categories of outdoor walkers, with significantly higher muscle scores in distal myotomes L4 (motor score median [range] = 4 [1-5]), L5 (motor score median [range] = 4 [1-5]), and S1 (motor score median [range] = 4 [2-5]) for the category independent of any aids. Further analyses of the 4 walking categories on myotome level for the LI leg ≤15 days after injury, and for the MI and LI leg at 6 months after injury are provided in eTables 4 to 6.

**Table 2.** Group Comparisons Between Categories of Walking Function: MI Leg ≤15 Days After Injury.

MI leg ≤15 days after injury	Non-walkers (N = 242)	Indoor walkers (N = 35)	Outdoor walkers with aids (N = 46)	Outdoor walkers without aids (N = 38)	Adjusted *P* value
L2	0 (0-1)	1 (0-5)			**<.001**
		1 (0-5)	2 (0-5)		.122
			2 (0-5)	4 (1-5)	.237
L3	0 (0-1)	2 (0-4)			**<.001**
		2 (0-4)	3 (0-5)		.159
			3 (0-5)	5 (2-5)	.173
L4	0 (0-0)	0 (0-4)			**.022**
		0 (0-4)	1 (0-4)		**.002**
			1 (0-4)	4 (1-5)	**<.001**
L5	0 (0-0)	0 (0-3)			.377
		0 (0-3)	1 (0-3)		**<.001**
			1 (0-3)	4 (1-5)	**<.001**
S1	0 (0-0)	0 (0-4)			.092
		0 (0-4)	1 (0-4)		**<.001**
			1 (0-4)	4 (2-5)	**<.001**

Abbreviations: L2, myotome of hip flexors; L3, myotome of knee extensors; L4, myotome of ankle dorsiflexors; L5, myotome of long toe extensors; LI, less impaired; MI, more impaired; N, number of patients; S1, myotome of ankle plantar flexors.

Comparison of the 4 categories of walking function at 6 months after injury (non-walkers, indoor walkers, outdoor walkers with aids, and outdoor walkers without aids) on group level (median [range]) for the 5 myotomes (L2–S1) of the MI leg ≤15 days after injury. Significant differences between groups (*P* < .05) are indicated in bold, tested by Bonferroni post hoc comparisons of Kruskal-Wallis tests. A comparison among the walking groups for the LI leg ≤15 days after injury, and for the MI and LI leg at 6 months after injury is provided in eTables 4-6.

Table 3 displays the number of patients from the study group with individual myotome motor scores greater than zero ≤15 days after injury and the proportion of these patients with walking function at 6 months after injury. Based on this, 85% of patients with a motor score in the L2 myotome of 1 or higher achieved ambulatory capacity throughout rehabilitation. For the L3 myotome the proportion was 88%. We also considered the motor scores (greater than 2) in individual myotomes at 6 months after injury and determined the proportion of patients being able to walk at this timepoint. More than 93% of patients with a motor score of 3 or higher in myotomes L2 or L3 were able to walk.

**Table 3.** Proportion of Walkers Depending on the Motor Score of Key Muscle Functions.

		L2		L3		L4		L5		S1	
15 days		≥1	=0	≥1	=0	≥1	=0	≥1	=0	≥1	=0
LEMS MI	N	119	242	115	246	73	288	69	292	74	287
Walkers (6 months)	N (%)	101 (85)	29 (12)	101 (88)	29 (12)	69 (95)	61 (22)	67 (98)	63 (22)	70 (95)	60 (21)
		≥1	=0	≥1	=0	≥1	=0	≥1	=0	≥1	=0
LEMS LI	N	131	230	121	240	89	272	83	278	84	277
Walkers (6 months)	N (%)	108 (82)	22 (10)	105 (87)	25 (10)	82 (92)	48 (18)	75 (90)	55 (20)	78 (93)	52 (19)
6 months		>2	≤2	>2	≤2	>2	≤2	>2	≤2	>2	≤2
LEMS MI	N	117	244	120	241	82	279	84	277	91	270
Walkers (6 months)	N (%)	109 (93)	21 (9)	113 (94)	17 (7)	79 (96)	51 (18)	80 (95)	50 (18)	87 (96)	43 (16)
		>2	≤2	>2	≤2	>2	≤2	>2	≤2	>2	≤2
LEMS LI	N	138	223	136	225	100	261	97	264	101	260
Walkers (6 months)	N (%)	118 (86)	12 (5)	120 (88)	10 (4)	93 (93)	37 (14)	89 (92)	41 (16)	93 (92)	37 (14)

Abbreviations: 6MWT, 6-minute walk test; L2, myotome of hip flexors; L3, myotome of knee extensors; L4, myotome of ankle dorsiflexors; L5, myotome of long toe extensors; LEMS, lower extremity motor score; LI, less impaired; MI, more impaired; N, number of patients; S1, myotome of ankle plantar flexors.

The number of patients from the study group (N = 361) with a corresponding LEMS value of the key muscles within myotomes L2–S1, for the MI and LI leg, ≤15 days (upper part of table) and 6 months (lower part of table) after injury is presented. Based on this, the number and proportion of patients who achieve to become ambulatory (based on the 6MWT at 6 months after injury) is reported.

**Figure 3.**
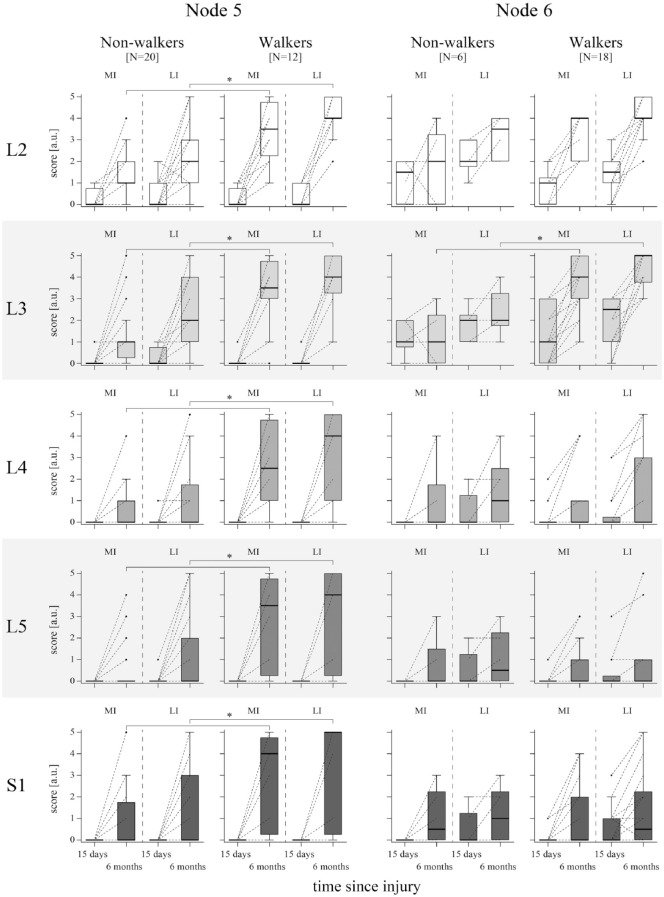
Neurological status and recovery within nodes 5 and 6 of the 6MWT URP–CTREE. The nodes are further split up into non-walkers and walkers (based on the 6MWT at 6 months after injury), respectively. LEMS is divided in its component parts (five myotomes on the spinal cord levels L2–S1). Key muscle function according to the myotomes is displayed for every node and divided into MI and LI leg at the timepoints ≤15 days and 6 months after injury (individual patient values are connected between the 2 timepoints with a dotted line). Data are shown as boxplots (whiskers: Tukey). Significant differences between walkers and non-walkers at 6 months after injury are indicated by asterisks (*P* < .05) tested by Mann–Whitney *U* tests with Bonferroni correction for multiple comparisons. Abbreviations: 6MWT, 6-minute walk test; a.u., arbitrary unit; L2, myotome of hip flexors; L3, myotome of knee extensors; L4, myotome of ankle dorsiflexors; L5, myotome of long toe extensors; LEMS, lower extremity motor score; LI, less impaired; MI, more impaired; N, number of patients; S1, myotome of ankle plantar flexors; URP–CTREE, unbiased recursive partitioning conditional inference tree.

Due to the heterogeneous composition of nodes 5 and 6 in terms of walking function (containing 38% and 75% of walkers, respectively), we investigated whether differences on myotome level were detectable between non-walkers and walkers in these nodes (Figure 3). No significant differences were found between groups at the timepoint ≤15 days after injury in both nodes. But at 6 months after injury, walkers in node 5 demonstrated significantly higher motor scores in all myotomes compared to non-walkers. In node 6, walkers exhibited a significantly higher muscle function only in the L3 myotome.

## Discussion

This study provides a method for stratification of a heterogeneous population of individuals with paraplegic SCI into more homogeneous subgroups. Our subsequent node-specific analysis further revealed decisive factors of walking function. We report the proximal myotomes of the MI leg, corresponding to the muscle functions of hip flexion and knee extension, being the primary determinants for ambulation in terms of their prognostic (≤15 days after injury) and characterizing (at 6 months after injury) value, particularly for the differentiation between walkers and non-walkers. These findings are in line with previous studies, emphasizing on the importance of L2 and L3 myotomes in specifying walking function.^17,18,34^ Further distinction of ambulation reveals that residual muscle function in distal myotomes L4–S1 of the MI leg (≤15 days after injury) is crucial to become an outdoor walker 6 months after injury. In general, the initial injury severity and recovery of the MI leg seems to be an important driver to determine the extent of restoration of walking function. In contrast to earlier findings, we provide a prognostic classification of walking function earlier after injury and into more functional categories.

Although the project’s scope only focuses on SCI, URP–CTREE is potentially applicable to further neurological disorders with heterogeneous clinical manifestations. The advantages of a lower variability in the identified subgroups and an accordingly more precise prediction of expected rehabilitation outcomes are of potential benefit for application in clinical trials, but also to provide additional information to patients and health professionals in clinical practice regarding the tailoring of rehabilitation. A variety of clinical prediction algorithms have been developed over the last years, targeting outcomes related to walking function.^7-12^ Until now, almost all studies considered either a dichotomous or ordinally scaled outcome, reporting good results in terms of prediction accuracy/sensitivity. However, by missing on the stratification of a continuous outcome, they seem insufficient in terms of identifying more homogeneous subgroups.^35^ Moreover, this may introduce bias into any subsequent analysis owing to the persistently higher outcome variability in the subgroups.

Recently, we reported a continuous outcome stratification for individuals with cervical SCI.^12^ In contrast to this work, the current study included individuals with paraplegic SCI. We did not include patients with tetraplegia into this analysis of walking function, because depending on the neurological level of injury, the upper extremities are either fully functional or (partially) impaired, introducing a bias with regard to the use of walking aids. The decision to perform separate analyses for patients with para- and tetraplegia is further supported by findings that showed generally deviating results in the accuracy of outcome prediction of walking function in patients with para- and tetraplegia.^7,16^

### Study Population

Study and reference groups presented a slightly divergent AIS grade distribution toward more severely impaired patients in the study group. However, the comparison of baseline predictors by AIS grade between the 2 groups resulted in similar distributions. Hence, we could demonstrate that the potential bias we introduced in the analysis only concerns the frequency in which patients with a particular injury severity occur but not their respective baseline characteristics. We therefore assume our results to be generalizable to the population of patients with paraplegic SCI in the EMSCI network. The EMSCI database itself has been compared previously with data from a North American clinical trial (Sygen),^36^ demonstrating similarities in motor recovery after sensorimotor complete and incomplete cervical SCI.^35,37^

### Stratification Model

The nodes of the URP–CTREE were defined by distinctive model-selected predictors. Among these, LEMS, representing the residual muscle function after acute SCI, appeared to be the most important predictor in terms of determining the outcome of 6MWT.^8,38^ The nodes with poorest and best stratified outcome were finally divided by LT. It is widely accepted that LT assesses the integrity of the dorsal column.^39^ Since proprioception is as well represented in these fibre tracts, we assume that deficits in LT, related to a poorer proprioception, lead to less recovery in walking function. Nodes 10 to 12, characterized by moderate ambulation function, were split according to age and PP. Age seems to be of certain importance to stratify moderate walking outcomes, since we observed this already in individuals with cervical SCI.^12^ Patients of older age, despite presented with comparable neurological status to younger patients, do not appear to translate this potential accordingly into functional recovery.^40^ PP sensation is a marker for spinothalamic tract function. The selection of preserved PP sensation as a split probably relies on the spatial proximity of the spinothalamic to the lateral corticospinal tract and the corresponding correlation to motor recovery.^41,42^ Furthermore, we assume that the sensory scores (LT and PP) also provide information encoding lesion level and injury severity, potentially contributing to their value as predictors to split the subpopulations. NLI was not selected as a significant predictor by the stratification model to divide the population, indicating that the level of injury, considered individually, is not a determining factor for walking function. This is likely because the NLI itself only provides information on the location (ie, height) of the injury and does not cover the severity (eg, completeness) of injury.

The proportion of non-walkers and walkers in the nodes reveals that 75% or more of patients allocated to nodes 6 and higher become ambulatory 6 months after injury, providing valuable information for the planning of rehabilitation regarding its main focus. For node 4 the proportion of walkers lies below 5%, suggesting a continuous evaluation of the potentially beneficial progress in the domain of walking. The categorization (based on the 10 mWT) of nodes into non-walkers, indoor, and outdoor walkers corresponds to the results of median values in SCIM III_12-14_ (representing whether and at what distances ambulation can be applied in daily life). This confirms that our classification of walking function is valid not only in a clinical setting, but also for the transfer into everyday life.

In SCI, classification of injury severity (AIS) is widely used as factor to divide a patient group into multiple subgroups. Considering our findings of a widespread distribution of patients representing AIS grades A-D in most of the nodes, the question is raised as to the value of AIS as predictor and its potential to lower variability of the targeted outcome in the subgroups.

### Lower Extremity Myotomes

Analysis of the five lower extremity myotomes across nodes revealed a gradient in muscle function from absent values in the poorest outcome node to excellent motor scores in the nodes with the best walking function ≤15 days and 6 months after injury. Proximal myotomes L2 and L3 were thereby found to be the primary factors to distinguish between non–walkers and walkers. On the one hand, they determine if ambulation will prospectively be achieved (threshold for L2 and L3 motor scores of 1 [palpable or visible contraction] or more in the MI leg ≤15 days after injury). On the other hand, they define the thresholds for being able to walk (threshold for L2 and L3 motor scores of 3 [allowing active movement, with full ROM against gravity] or more in the MI leg at 6 months after injury).^15-18,34^ We primarily considered the MI leg rather than the LI leg regarding the categorization of walking function, because owing to the ordinal scale of the LEMS the results obtained for the LI leg are more likely to exhibit a ceiling effect.

While motor scores of proximal myotomes allow in most cases the distinction between walkers and non-walkers, muscle functions at the distal levels L4–S1 differentiate between indoor walkers (relying on a wheelchair outdoors) and those ambulating outdoors (with and without aids). This indicates that motor scores of distal myotomes rather determine the walking distance and the extent to which the regained walking function can be transferred into everyday life, than whether patients will walk or not. This is to a certain extent in contrast to earlier findings, which introduced either a combination of L3 and L5 or S1 to predict the general ability to walk.^8,19^ Unlike in our study, other studies assessed walking function with a dichotomous outcome, selecting SCIM III_12_ and SCIM III_14_ as respective endpoints related to walking function. Stratification based on a dichotomous endpoint poses the risk of obtaining rather heterogeneous subgroups (eg, when all walkers are grouped together), affecting further analyses. This and the fact that van Middendorp et al^8^ included both patients with paraplegia and tetraplegia potentially explain the discrepancy to our results.

We did not find any difference in myotomes ≤15 days after injury in walkers and non–walkers subgroups of nodes 5 and 6. These findings point out that we are, with the current baseline assessments selected, not able to reliably stratify the walking outcome in these subgroups. The heterogeneity in outcome present in this subpopulation, although similar baseline characteristics, is based on diverging recovery patterns. These discrepancies may either originate from secondary complications potentially affecting recovery or insufficiently sensitive assessments. The latter emphasizes the need of the inclusion of additional, more sensitive assessments (eg, neurophysiological examinations or MRI measurements) acutely after injury, to enable a more reliable prognosis of recovery.^7^

### Limitations

The patient population (study and reference groups) extracted from the EMSCI database was selected based on completeness of data. This may have introduced bias, as data availability varies with the severity of injury (observed in a higher frequency of AIS A patients in the study group). The predictors we considered for the stratification model are routinely assessed in clinical practice, leading to likely higher numbers of complete datasets. However, the simplicity of these measures may result in missing out on information, which could be captured by more complex assessments. The EMSCI study protocol does not cover all variables (eg, secondary complications or therapy interventions) influencing predictors and outcomes, potentially interfering with the accuracy of the stratification model.

## Conclusion

URP–CTREE enables the stratification of a heterogeneous population with paraplegic SCI into more homogeneous subgroups, with potential benefit for the application in clinical trials, allowing to tailor treatments to the needs of these subgroups and select the appropriate study participants. Moreover, this method may provide additional useful information on expected rehabilitation outcome to patients and health professionals. Based on the identified subgroups, proximal lower extremity myotomes L2 and L3 of the MI leg were found to determine best which patients will prospectively recover walking function as well as which thresholds must be met to be able to walk. Future studies are needed to further improve stratification and prediction of patients with SCI based on current assessments, as well as to explore the implementation of promising new ones.

## Supplemental Material

sj-pdf-1-nnr-10.1177_15459683231166937 – Supplemental material for Walking Outcome After Traumatic Paraplegic Spinal Cord Injury: The Function of Which Myotomes Makes a Difference? Click here for additional data file. Supplemental material, sj-pdf-1-nnr-10.1177_15459683231166937 for Walking Outcome After Traumatic Paraplegic Spinal Cord Injury: The Function of Which Myotomes Makes a Difference? by Adrian Cathomen, Doris Maier, Jiri Kriz, Rainer Abel, Frank Röhrich, Michael Baumberger, Giorgio Scivoletto, Norbert Weidner, Rüdiger Rupp, Catherine R. Jutzeler, John D. Steeves, Armin Curt and Marc Bolliger in Neurorehabilitation and Neural Repair
