# Supplementary material for: Walking Outcome After Traumatic Paraplegic Spinal Cord Injury: The Function of Which Myotomes Makes a Difference?
Source: Neurorehabil Neural Repair. 2023 Apr 11;37(5):316–27. doi: 10.1177/15459683231166937 (PMC10272624; doi:10.1177/15459683231166937)
Supplement: sj-pdf-1-nnr-10.1177_15459683231166937 – Supplemental material for Walking Outcome After Traumatic Paraplegic Spinal Cord Injury: The Function of Which Myotomes Makes a Difference? [file sj-pdf-1-nnr-10.1177_15459683231166937.pdf]

Supplemental Material

eMethods

Data sanity checks

eFigures

eFigure 1. Flow chart of patient numbers

eFigure 2. Comparison of baseline LEMS, LT, and PP between the study and reference groups divided into AIS grades

eFigure 3. Neurological status and recovery within the nodes of the 6MWT URP–CTREE (extended version)

eTables

eTable 1. American Spinal Injury Association impairment scale (AIS)

eTable 2. Lower extremity motor score: motor examination of key muscle functions of corresponding myotomes

eTable 3. NLI and AIS grades within the nodes of the 6MWT URP–CTREE

eTable 4. Group comparisons between categories of walking function: LI leg ≤15 days after injury

eTable 5. Group comparisons between categories of walking function: MI leg at 6 months after injury

eTable 6. Group comparisons between categories of walking function: LI leg at 6 months after injury

eMethods

Data sanity checks

All EMSCI centres entered their data on site. After upload to the main database in a pseudonymised form, the central EMSCI database manager verified the data. A conservative sanity test was performed on the extracted datasets. A data point was assigned inconclusive (N=28) if its value was found to be contradictory compared to related values of measures for the same subject at the same timepoint. This was evaluated by manual inspection and automated application of a series of predefined rules at the timepoint 6 months after injury. Data violating any of the following rules were excluded from further analysis.

1. SCIM $III_{12–14} > 9$ and distance in 6MWT = 0 m.
2. SCIM $III_{12–14} > 9$ and speed in 10mWT = 0 m/s.
3. Distance in 6MWT = 0 m and speed in 10mWT > 0 m/s, and vice versa.

The maximal score of the SCIM $III_{12–14}$ is 24 points. The threshold of nine points (three points in every item) was set in an attempt at a conservative approach to define the worst possible clinical condition in which the accomplishment of a 6MWT and 10mWT is still feasible, implying expected values greater than zero.

eFigure 1. Flow chart of patient numbers

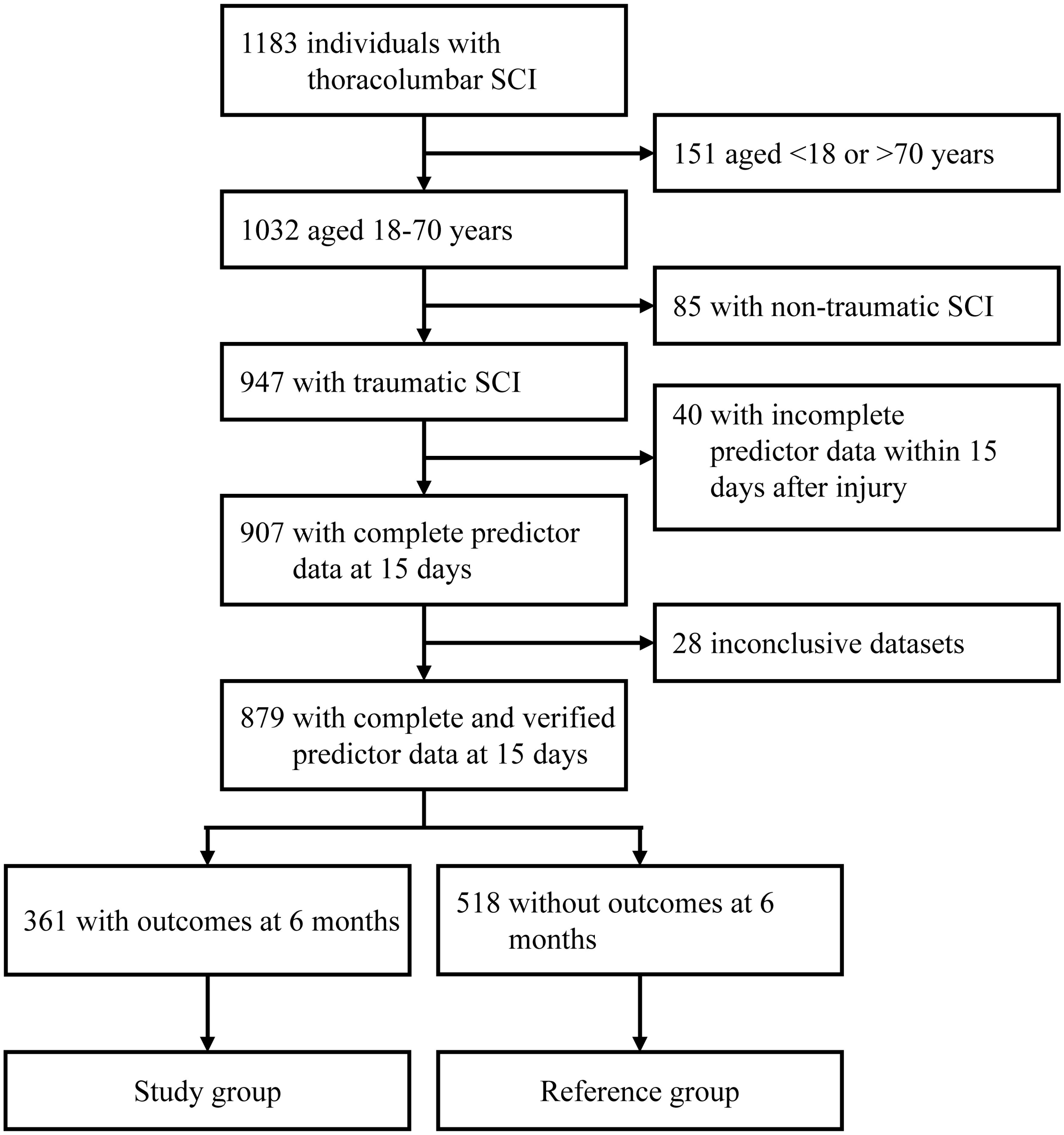

Selection criteria used for data presented here, extracted from the EMSCI database on 19th February 2020. We defined NLI, LEMS, LT, PP, and age as predictors ≤15 days after injury. Outcomes at 6 months after injury included 6MWT, 10mWT, SCIM III12-14, WISCI II, and LEMS. The allocation of participants to the study or reference groups was based on the criterion of completeness of outcome data at 6 months after injury. Participants with complete outcome data were assigned to the study group, whereas participants with incomplete outcome data were assigned to the reference group. The study group was used to develop the stratification model and subsequent analyses. With the reference group the representativity of the study group was analysed. 6MWT, 6–minute walk test; 10mWT, 10–meter walk test; EMSCI, European Multicenter Study about Spinal Cord Injury; LEMS, lower extremity motor score; LT, light touch; NLI, neurological level of injury; PP, pin prick; SCI, spinal cord injury; SCIM III12-14, spinal cord independence measure III items 12-14; WISCI II, walking index for spinal cord injury.

eFigure 2. Comparison of baseline LEMS, LT, and PP between the study and reference groups divided into AIS grades

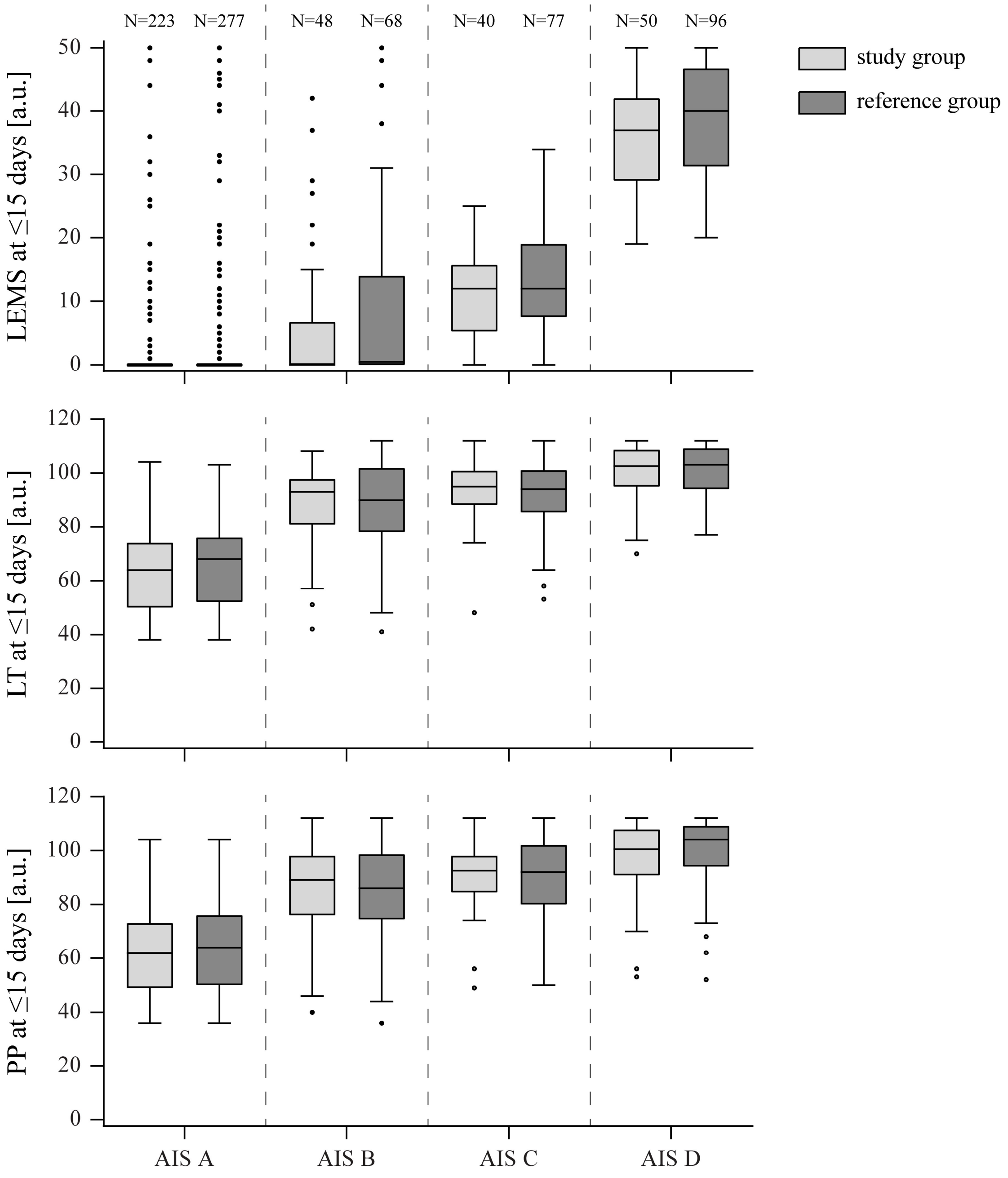

Comparison of median LEMS, LT, and PP scores ≤15 days after injury between the study and reference groups by initial AIS grade (A–D). Mann–Whitney–U tests for non–parametric independent data, corrected for multiple comparisons (Bonferroni), were performed to test for significant differences in LEMS, LT, and PP between groups. No significant differences were observed (significance level $p<0.05$). AIS, American Spinal Injury Association impairment scale; a.u., arbitrary unit; LEMS, lower extremity motor score; LT, light touch; N, number of patients; PP, pin prick.

eFigure 3. Neurological status and recovery within the nodes of the 6MWT URP–CTREE (extended version)

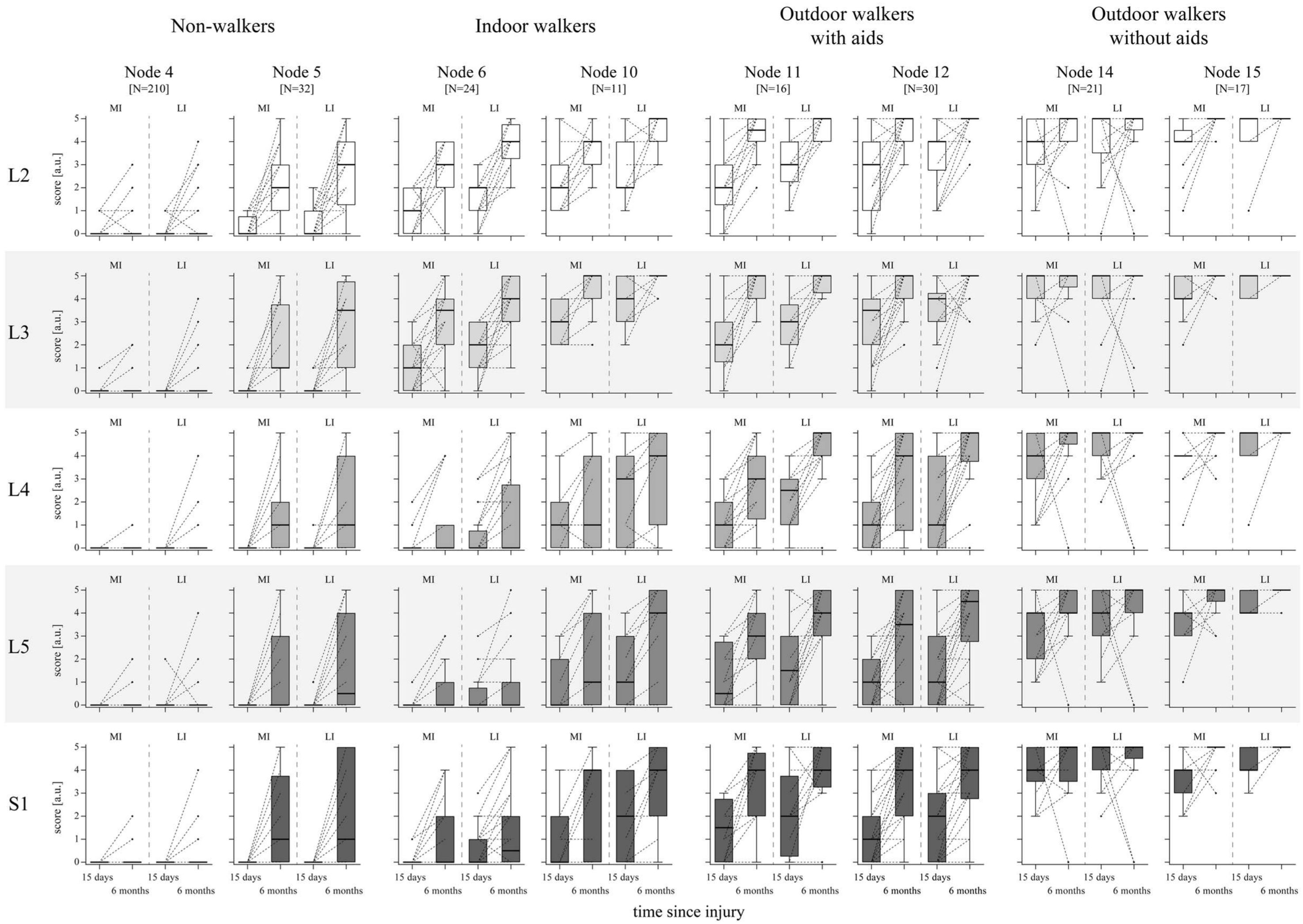

LEMS is divided in its component parts (five myotomes on the spinal cord levels L2-S1). Key muscle function according to the myotomes is displayed for every node and divided into MI and LI leg at the timepoints ≤15 days and 6 months after injury (individual patient values are connected between the two timepoints with a dotted line). Data are shown as boxplots (whiskers: Tukey) for the categories of non–walkers (nodes 4 and 5), indoor walkers (nodes 6 and 10), and outdoor walkers (nodes 11, 12, 14, and 15). 6MWT, 6–minute walk test; a.u., arbitrary unit; L2, myotome of hip flexors; L3, myotome of knee extensors; L4, myotome of ankle dorsiflexors; L5, myotome of long toe extensors; LEMS, lower extremity motor score; LI, less impaired; MI, more impaired; N, number of patients; S1, myotome of ankle plantar flexors; URP–CTREE, unbiased recursive partitioning conditional inference tree.

eTable 1. American Spinal Injury Association impairment scale (AIS)

Grades	Description
A	No sensory or motor function is preserved in the sacral segments S4-5.
B	Sensory but not motor function is preserved below the neurological level and includes the sacral segments S4-5 (light touch or pin prick at S4-5 or deep anal pressure) AND no motor function is preserved more than three levels below the motor level on either side of the body.
C	Motor function is preserved at the most caudal sacral segments for voluntary anal contraction (VAC) OR the patient meets the criteria for sensory incomplete status (sensory function preserved at the most caudal sacral segments S4-5 by LT, PP or DAP), and has some sparing of motor function more than three levels below the ipsilateral motor level on either side of the body. (This includes key or non-key muscle functions to determine motor incomplete status.) For AIS C – less than half of key muscle functions below the single NLI have a muscle grade ≥ 3.
D	Motor incomplete status as defined above, with at least half (half or more) of key muscle functions below the single NLI having a muscle grade ≥ 3.
E	If sensation and motor function as tested with the ISNCSCI are graded as normal in all segments, and the patient had prior deficits, then the AIS grade is E. Someone without an initial SCI does not receive an AIS grade.

Classification of injury severity (i.e. completeness) for individuals with SCI.

eTable 2. Lower extremity motor score: motor examination of key muscle functions of corresponding myotomes

Myotomes & key muscles		Grades of muscle function	
L2	Hip flexors (*m. iliopsoas*)	0	total paralysis
L3	Knee extensors (*m. quadriceps*)	1	palpable or visible contraction
L4	Ankle dorsiflexors (*m. tibialis anterior*)	2	active movement, full ROM with gravity eliminated
L5	Long toe extensors (*m. extensor hallucis longus*)	3	active movement, full ROM against gravity
S1	Ankle plantar flexors (*m. gastrocnemius, m. soleus*)	4	active movement, full ROM against gravity and moderate resistance in a muscle specific position
		5	(normal) active movement, full ROM against gravity and full resistance in a muscle specific position expected from an otherwise unimpaired person

Determination of lower extremity muscle function using the LEMS assessment. Key muscles within the myotomes on the spinal cord levels L2-S1 are assessed in terms of muscle function. m., musculus; ROM, range of motion.

eTable 3. Neurological level of injury and AIS grades within the nodes of the 6MWT URP–CTREE

		Node 4 (N=210)		Node 5 (N=32)		Node 6 (N=24)		Node 10 (N=11)		Node 11 (N=16)		Node 12 (N=30)		Node 14 (N=21)		Node 15 (N=17)	
**Neurological level of injury**																	
15 days		N	%	N	%	N	%	N	%	N	%	N	%	N	%	N	%
	T2-12	208	99.0	24	75.0	15	62.5	4	36.4	11	68.7	13	43.3	14	66.7	2	11.8
	L1-5	2	1.0	8	25.0	9	37.5	7	63.6	5	31.3	17	56.7	7	33.3	15	88.2
**AIS grades**																	
15 days		N	%	N	%	N	%	N	%	N	%	N	%	N	%	N	%
	A	195	92.9	7	21.9	6	25.0	1	9.1	8	50.0	2	6.7	4	19.1	0	0.0
	B	12	5.7	19	59.4	9	37.5	1	9.1	0	0.0	5	16.7	2	9.5	0	0.0
	C	3	1.4	6	18.8	9	37.5	6	54.6	4	25.0	12	40.0	0	0.0	0	0.0
	D	0	0.0	0	0.0	0	0.0	3	27.3	4	25.0	11	36.7	15	71.4	17	100.0
	E	0	0.0	0	0.0	0	0.0	0	0.0	0	0.0	0	0.0	0	0.0	0	0.0
6 months																	
	A	178	84.8	4	12.5	1	4.2	1	9.1	5	31.3	1	3.3	4	19.1	0	0.0
	B	17	8.1	8	25.0	5	20.8	0	0.0	0	0.0	1	3.3	1	4.8	0	0.0
	C	14	6.7	10	31.3	12	50.0	2	18.2	0	0.0	4	13.3	0	0.0	0	0.0
	D	1	0.5	10	31.3	6	25.0	8	72.7	11	68.8	24	80.0	15	71.4	11	64.7
	E	0	0.0	0	0.0	0	0.0	0	0.0	0	0.0	0	0.0	1	4.8	6	35.3

Neurological level of injury (at ≤15 days) and severity of injury (at ≤15 days and 6 months) are presented for the nodes of the 6MWT URP–CTREE. AIS, American Spinal Injury Association impairment scale; N, number of patients; URP–CTREE, unbiased recursive partitioning conditional inference tree.

eTable 4. Group comparisons between categories of walking function: LI leg ≤15 days after injury

LI leg ≤15 days after injury	Non-walkers [N=242]	Indoor walkers [N=35]	Outdoor walkers with aids [N=46]	Outdoor walkers without aids [N=38]	Adjusted p-value
L2	0 (0-2)	2 (0-5)			**<0.001**
		2 (0-5)	3 (1-5)		0.281
			3 (1-5)	5 (0-5)	1.000
L3	0 (0-1)	3 (0-5)			**<0.001**
		3 (0-5)	4 (0-5)		1.000
			4 (0-5)	5 (0-5)	0.566
L4	0 (0-1)	0 (0-5)			**<0.001**
		0 (0-5)	2 (0-5)		**0.002**
			2 (0-5)	5 (1-5)	**<0.001**
L5	0 (0-2)	0 (0-4)			**<0.001**
		0 (0-4)	1 (0-5)		**0.019**
			1 (0-5)	4 (1-5)	**<0.001**
S1	0 (0-0)	0 (0-4)			**<0.001**
		0 (0-4)	2 (0-5)		**0.035**
			2 (0-5)	4.5 (2-5)	**<0.001**

Comparison of the four categories of walking function at 6 months after injury (non-walkers, indoor walkers, outdoor walkers with aids, and outdoor walkers without aids) on group level (median [range]) for the five myotomes (L2-S1) of the LI leg ≤15 days after injury. Significant differences between groups ($p<0.05$) are indicated in bold, tested by Bonferroni post hoc comparisons of Kruskal-Wallis tests. L2, myotome of hip flexors; L3, myotome of knee extensors; L4, myotome of ankle dorsiflexors; L5, myotome of long toe extensors; LI, less impaired; N, number of patients; S1, myotome of ankle plantar flexors.

eTable 5. Group comparisons between categories of walking function: MI leg at 6 months after injury

MI leg<br>6 months after injury	Non-walkers<br>[N=242]	Indoor walkers<br>[N=35]	Outdoor walkers with aids<br>[N=46]	Outdoor walkers without aids<br>[N=38]	Adjusted p-value
L2	0 (0-5)	4 (0-5)			**<0.001**
		4 (0-5)	5 (2-5)		0.070
			5 (2-5)	5 (0-5)	1.000
L3	0 (0-5)	4 (0-5)			**<0.001**
		4 (0-5)	5 (2-5)		0.532
			5 (2-5)	5 (0-5)	1.000
L4	0 (0-5)	0 (0-5)			**0.001**
		0 (0-5)	3 (0-5)		**0.002**
			3 (0-5)	5 (0-5)	**0.036**
L5	0 (0-5)	0 (0-5)			**0.002**
		0 (0-5)	3 (0-5)		**<0.001**
			3 (0-5)	5 (0-5)	**0.041**
S1	0 (0-5)	1 (0-5)			**<0.001**
		1 (0-5)	4 (0-5)		**0.001**
			4 (0-5)	5 (0-5)	0.303

Comparison of the four categories of walking function at 6 months after injury (non-walkers, indoor walkers, outdoor walkers with aids, and outdoor walkers without aids) on group level (median [range]) for the five myotomes (L2-S1) of the MI leg at 6 months after injury. Significant differences between groups ($p<0.05$) are indicated in bold, tested by Bonferroni post hoc comparisons of Kruskal-Wallis tests. L2, myotome of hip flexors; L3, myotome of knee extensors; L4, myotome of ankle dorsiflexors; L5, myotome of long toe extensors; MI, more impaired; N, number of patients; S1, myotome of ankle plantar flexors.

eTable 6. Group comparisons between categories of walking function: LI leg at 6 months after injury

LI leg 6 months after injury	Non-walkers [N=242]	Indoor walkers [N=35]	Outdoor walkers with aids [N=46]	Outdoor walkers without aids [N=38]	Adjusted p-value
L2	0 (0-5)	4 (2-5)			**<0.001**
		4 (2-5)	5 (3-5)		0.727
			5 (3-5)	5 (0-5)	1.000
L3	0 (0-5)	5 (1-5)			**<0.001**
		5 (1-5)	5 (3-5)		1.000
			5 (3-5)	5 (0-5)	1.000
L4	0 (0-5)	1 (0-5)			**<0.001**
		1 (0-5)	5 (0-5)		**0.001**
			5 (0-5)	5 (0-5)	1.000
L5	0 (0-5)	1 (0-5)			**<0.001**
		1 (0-5)	4 (0-5)		**0.001**
			4 (0-5)	5 (0-5)	0.800
S1	0 (0-5)	2 (0-5)			**<0.001**
		2 (0-5)	4 (0-5)		**0.007**
			4 (0-5)	5 (0-5)	0.825

Comparison of the four categories of walking function at 6 months after injury (non-walkers, indoor walkers, outdoor walkers with aids, and outdoor walkers without aids) on group level (median [range]) for the five myotomes (L2-S1) of the LI leg at 6 months after injury. Significant differences between groups ($p<0.05$) are indicated in bold, tested by Bonferroni post hoc comparisons of Kruskal-Wallis tests. L2, myotome of hip flexors; L3, myotome of knee extensors; L4, myotome of ankle dorsiflexors; L5, myotome of long toe extensors; LI, less impaired; N, number of patients; S1, myotome of ankle plantar flexors.
